# Transcriptome analysis of *Brassica napus* pod using RNA-Seq and identification of lipid-related candidate genes

**DOI:** 10.1186/s12864-015-2062-7

**Published:** 2015-10-24

**Authors:** Hai-Ming Xu, Xiang-Dong Kong, Fei Chen, Ji-Xiang Huang, Xiang-Yang Lou, Jian-Yi Zhao

**Affiliations:** Institute of Bioinformatics, College of Agriculture and Biotechnology, Zhejiang University, Hangzhou, China; State Key Laboratory Breeding Base for Zhejiang Sustainable Pest and Disease Control, Institute of Crop and Nuclear Technology Utilization, Zhejiang Academy of Agricultural Sciences, 198 Shiqiao Road, Hangzhou, 310021 China; Department of Biostatistics and Bioinformatics, Tulane University, 1440 Canal St., Suite 2001, New Orleans, LA 70112-2632 USA

**Keywords:** Transcriptome, RNA-Seq, QTL regions, Comparative genomics, Lipid-related candidate genes, *Brassica napus*

## Abstract

**Background:**

*Brassica napus* is an important oilseed crop. Dissection of the genetic architecture underlying oil-related biological processes will greatly facilitates the genetic improvement of rapeseed. The differential gene expression during pod development offers a snapshot on the genes responsible for oil accumulation in. To identify candidate genes in the linkage peaks reported previously, we used RNA sequencing (RNA-Seq) technology to analyze the pod transcriptomes of German cultivar Sollux and Chinese inbred line Gaoyou.

**Methods:**

The RNA samples were collected for RNA-Seq at 5-7, 15-17 and 25-27 days after flowering (DAF). Bioinformatics analysis was performed to investigate differentially expressed genes (DEGs). Gene annotation analysis was integrated with QTL mapping and *Brassica napus* pod transcriptome profiling to detect potential candidate genes in oilseed.

**Results:**

Four hundred sixty five and two thousand, one hundred fourteen candidate DEGs were identified, respectively, between two varieties at the same stages and across different periods of each variety. Then, 33 DEGs between Sollux and Gaoyou were identified as the candidate genes affecting seed oil content by combining those DEGs with the quantitative trait locus (QTL) mapping results, of which, one was found to be homologous to *Arabidopsis thaliana* lipid-related genes.

**Discussion:**

Intervarietal DEGs of lipid pathways in QTL regions represent important candidate genes for oil-related traits. Integrated analysis of transcriptome profiling, QTL mapping and comparative genomics with other relative species leads to efficient identification of most plausible functional genes underlying oil-content related characters, offering valuable resources for bettering breeding program of *Brassica napus*.

**Conclusions:**

This study provided a comprehensive overview on the pod transcriptomes of two varieties with different oil-contents at the three developmental stages.

**Electronic supplementary material:**

The online version of this article (doi:10.1186/s12864-015-2062-7) contains supplementary material, which is available to authorized users.

## Background

Rapeseed (*Brassica napus* L.) is one of the primary sources of vegetable oil for human nutrition; it occupies a pivotal position on oil supply in China. Rapeseed production has remarkably increased as a result of the popularization of elite cultivars with high yield and good quality, and molecular design breeding has become one of the most available breeding methods. Dissection of the genetic architecture underlying the important agronomic traits will greatly facilitate the genetic improvement of rapeseed.

*Brassica napus* is an allopolyploid species derived from the natural interspecies hybridization between *Brassica rapa* and *Brassica oleracea*, the former contributing the A genome and the latter contributing the C genome. The seed oil content is a very important economic character in rapeseed production. And its genetic architecture has been investigated by quantitative trait locus (QTL) mapping based on appropriate mapping populations. Several QTLs controlling seed oil content have been identified [1]. Qiu et al. [2] analyzed the QTLs of seed oil and erucic acid content using a comparative linkage map of oilseed rape. Recently, Zhao et al. [3] detected eight QTLs with additive effects and nine pairs of loci with additive-additive epistasis. However, the resolution is still not enough to pinpoint the candidate genes for the surveyed trait because of too many genes in the identified QTL regions.

Systematic integration of genetic mapping with transcription and genome annotations is considered as a promising avenue to identify causal genes [4]. The differentially expressed genes are potential contributors to the difference between individuals. With rapid advance in biotechnology, the subtractive suppression hybridization (SSH) has been used to investigate differentially expressed genes underlying oil content of rapeseed [5]. Some important genes, such as yeast glycerol-3-phosphate dehydrogenase, Arabidopsis FAE1 and yeast SLC1-1 genes, have been suggested for use in the genetic improvement of the oil content of rapeseed [6, 7]. However, this technology has a few drawbacks, such as low resolution and low dynamic range of expression [8] and the relevant studies cannot explore integrated molecular mechanism underlying seed oil content. RNA sequencing (RNA-Seq) that uses deep-sequencing technologies, as an approach to transcriptome profiling, possess several advantages over the other expression profiling technologies, such as higher sensitivity and the ability to detect splicing isoforms and somatic mutations [9]. Transcriptome sequencing can provide massive sequence data for analysis of gene expression. Transcriptome sequencing has been applied in studies on *Brassica napus*, such as investigating single nucleotide polymorphism (SNP) [10], and measuring the contributions of homologues to gene expression [11]. However, little effort is being expended in attempts to investigate oil-related biological processes using RNA-Seq. Given that we have been able to dissect genetic architecture of oil content into several chromosomal loci by QTL mapping, the combined use of the QTL mapping with transcriptome profiling represents a practical solution to further refine the mapping resolution and identify potential candidate genes [12].

Here, we integrated QTL mapping and *Brassica napus* pod transcriptome profiling to detect potential candidate genes in oilseed. As rapeseed oil is accumulated during the development of pod and the differential expression in the parents offers a snapshot on that of the mapping population, in this study, we focused our research on the German variety Sollux and Chinese variety Gaoyou, which have been used as the parents to develop the QTL mapping population for oil content [3, 13]. We applied the RNA-Seq technique to characterize gene expression of *Brassica napu* pod at the growth stages of 5–7, 15–17 and 25–27 days after flowering (DAF). Bioinformatics analysis was performed to investigate candidate differentially expressed genes (DEGs or candidate DEGs) and their expression patterns. Intervarietal DEGs of lipid pathways were found to help us understand oil content difference between the two varieties. More importantly, DEGs involved in QTL regions are important candidate genes for breeding program especially those DEGs associated with lipid metabolism. Our study provides information on the growth of pod at the molecular level, identifies candidate genes responsible for oil content, and is also an application case of quantitative genetics integrated with next-generation sequencing (NGS) analysis and comparative genomics that have broad application prospects.

## Results

### Gene differential expression analysis

After the quality control, approximately 37.9 million pair-end reads (100-bp in size) and 26.1 million single-end reads (50-bp in size) were generated. On average of 82.8 % of reads were mapped to the reference genome by the Tophat (v2.0.12). After the annotation merging by cuffmerge, 157,229 genes were annotated, within which, 101,040 genes have been annotated with the publishing of the reference genome. Meanwhile, 185,965 different transcripts were identified in the merged annotation.

**Fig. 1:**
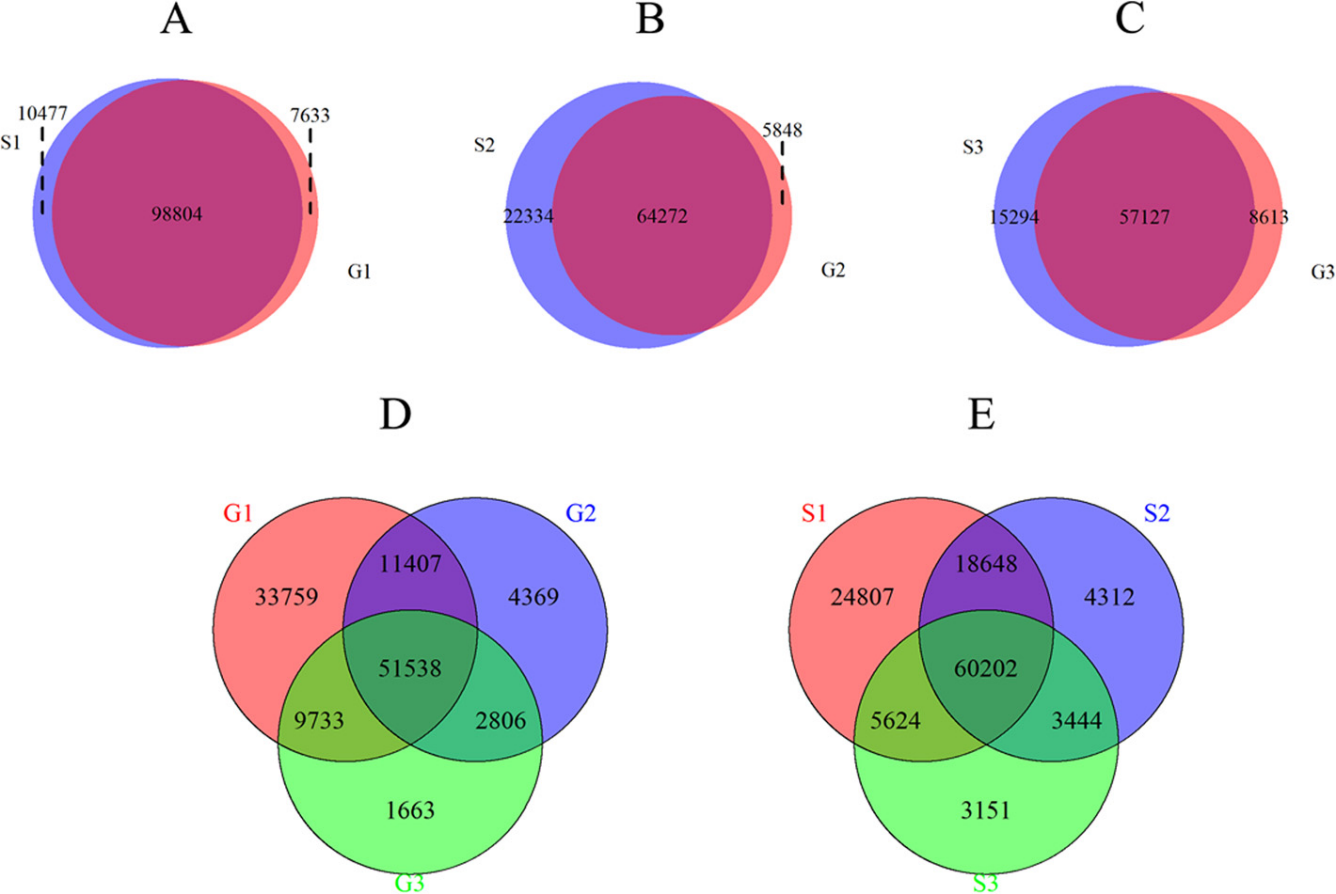
Venn diagram for the genes expressed in each of the three stages of *Brassica napus* pod in Sollux and Gaoyou. **a** 98,804 genes are co-expressed at S1 and G1 while 10,477 (S1) and 7,633 (G1) are variety-specifically expressed genes. **b** 64,272 genes are co-expressed at S2 and G2 while 22,334 (S2) and 5,848 (G2) are variety-specifically expressed genes. **c** 57,127 genes are co-expressioned at S3 and G3 while 15,294 (S3) and 8,613 (G3) are variety-specifically expressed genes. **d** Among all genes, 51,538 are co-expressed at all the stages in the Gaoyou, 11,407 are co-expressed in G1 and G2. 2,806 are co-expressed in G3 and G2, and 9,733 are co-expressed in G1 and G3. The numbers of stage-specifically expressed genes are 33,759 (G1), 4,396 (G2), and 1,663 (G3), respectively. **e** Among all the genes, 60,202 are co-expressed at all the stages in the Sollux, 18,648 are co-expressed in S1 and S2, 3,444 are co-expressed in S3 and S2, and 5,624 are co-expressed in S1 and S3. The numbers of stage-specifically expressed genes are 24,807 (S1), 4,312 (S2), and 3,151 (S3), respectively

Based on the gene expression levels calculated by FPKM (Fragments Per Kilobase of transcript per Million mapped reads), we found a close overlapping between stages and varieties. On one hand, 98,804, 64,272 and 57,127 co-expressed genes of the varieties were observed in three different stages, S1-G1, S2-G2 and S3-G3 respectively (Fig. 1a, b, c). On the other hand, in all three stages, 51,538 genes expressed in the Gaoyou and 60,202 genes expressed in the Sollux.

**Fig. 2:**
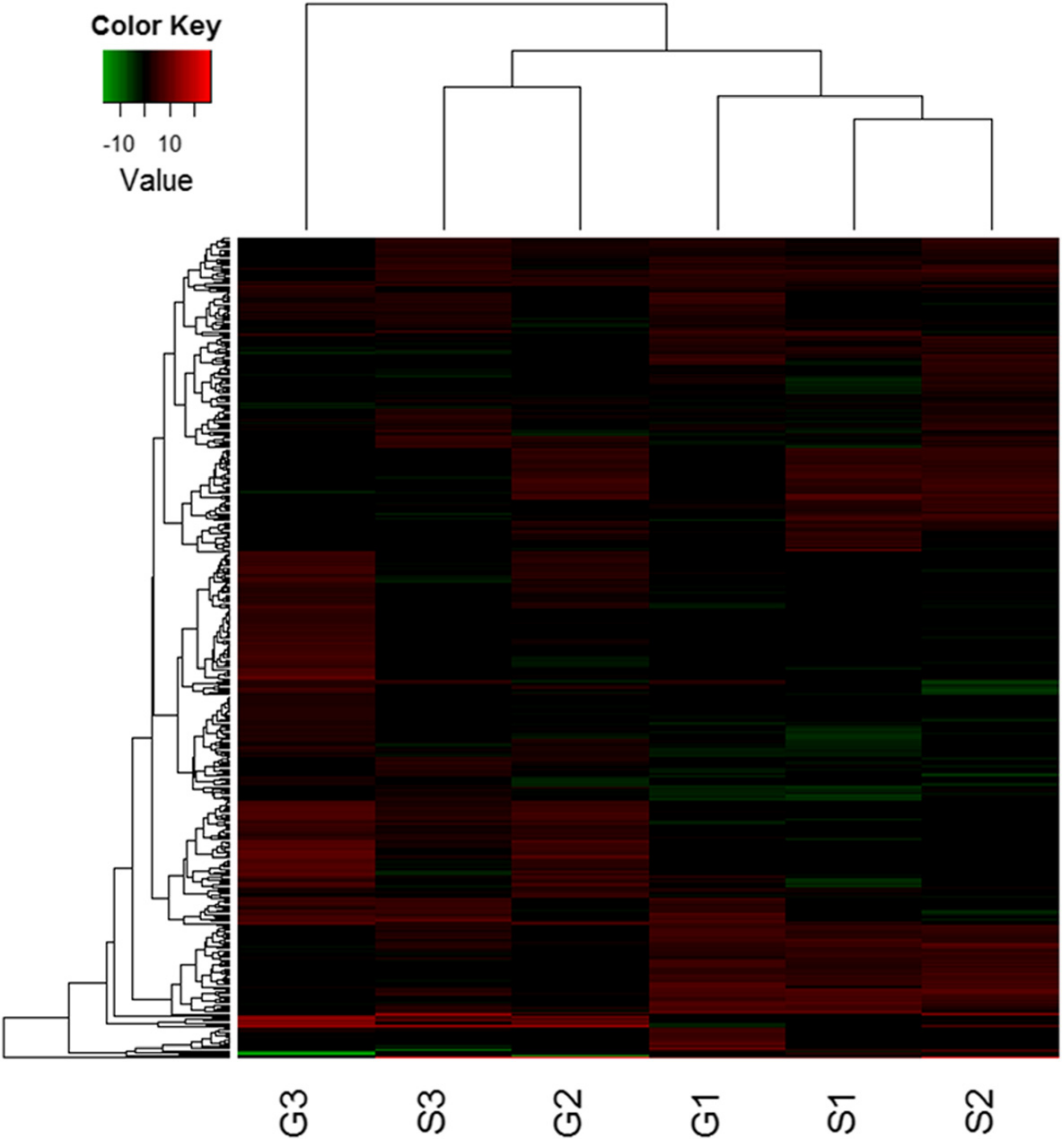
Hierarchical clustering analysis of gene expression based on RPKM data. S1, S2 and S3 are stages 1, 2 and 3, respectively for Sollux, and G1, G2 and G3 are stages 1, 2 and 3, respectively, for Gaoyou. The color key represents RPKM (reads per kilobase per million mapped reads) normalized log_2_ transformed counts. Red represents high expression and green represents low expression. Each row represents a gene

According to the criteria to determine differential expression of gene (FDR <0.05 and the p value < 0.01) [14], 465 candidate DEGs between Gaoyou and Sollux at the three stages were identified (Table 1). There was some difference in percentage of up-regulated and down-regulated DEGs between the two varieties. At 5–7 DAF, 96 DEGs were identified. The number of lowly expressed genes in Gaoyou (52.1 %) was larger than that in Sollux. At 15–17 DAF, the number of DEGs was 175, and 53.7 % of them exhibited lower expression in Gaoyou. The percentage of higher expression genes was 58.8 % in Gaoyou at 25–27 DAF. The numbers of up-regulated and down-regulated genes across different stages in Sollux and Gaoyou were presented in Table 2. More genes differentially expressed were up-regulated in the early growth stage than in the late stage for the Gaoyou, but it was diametrically opposite for the Sollux, indicating that the Gaoyou had more active gene expression in the early stage, while the Sollux had more in the late stage. Heat map from the hierarchical clustering of DEGs is shown in Fig. 2. The aforementioned genes up-regulated and down-regulated between varieties and/or stages were indicated by hierarchical clustering analysis.

**Table 1:** Number and classification of DEGs between the two varieties at the three stages

	S1-G1	S2-G2	S3-G3
Up-regulated	46	81	114
Down-regulated	50	94	80

S and G denote the Sollux and the Gaoyou, respectively. The numbers 1–3 denote the three stages, respectively. Up-regulated and down-regulated mean that the expression level in the Gaoyou is higher and lower than that in Sollux, respectively. The DEGs were defined by the criteria of FDR < 0.05, and the p value < 0.01

**Table 2:** Number and classification of DEGs between different stages for each variety

		1-2 (stage)	2-3 (stage)	1-3 (stage)	total
Sollux	up-regulated	118	275	446	839
	down-regulated	38	346	125	509
Gaoyou	up-regulated	451	415	384	1250
	down-regulated	144	331	136	611

Up-regulated and down-regulated, respectively, mean the expression level of the later stage is higher and lower than that of the former stage

### Functional classification by Gene Ontology

**Fig. 3:**
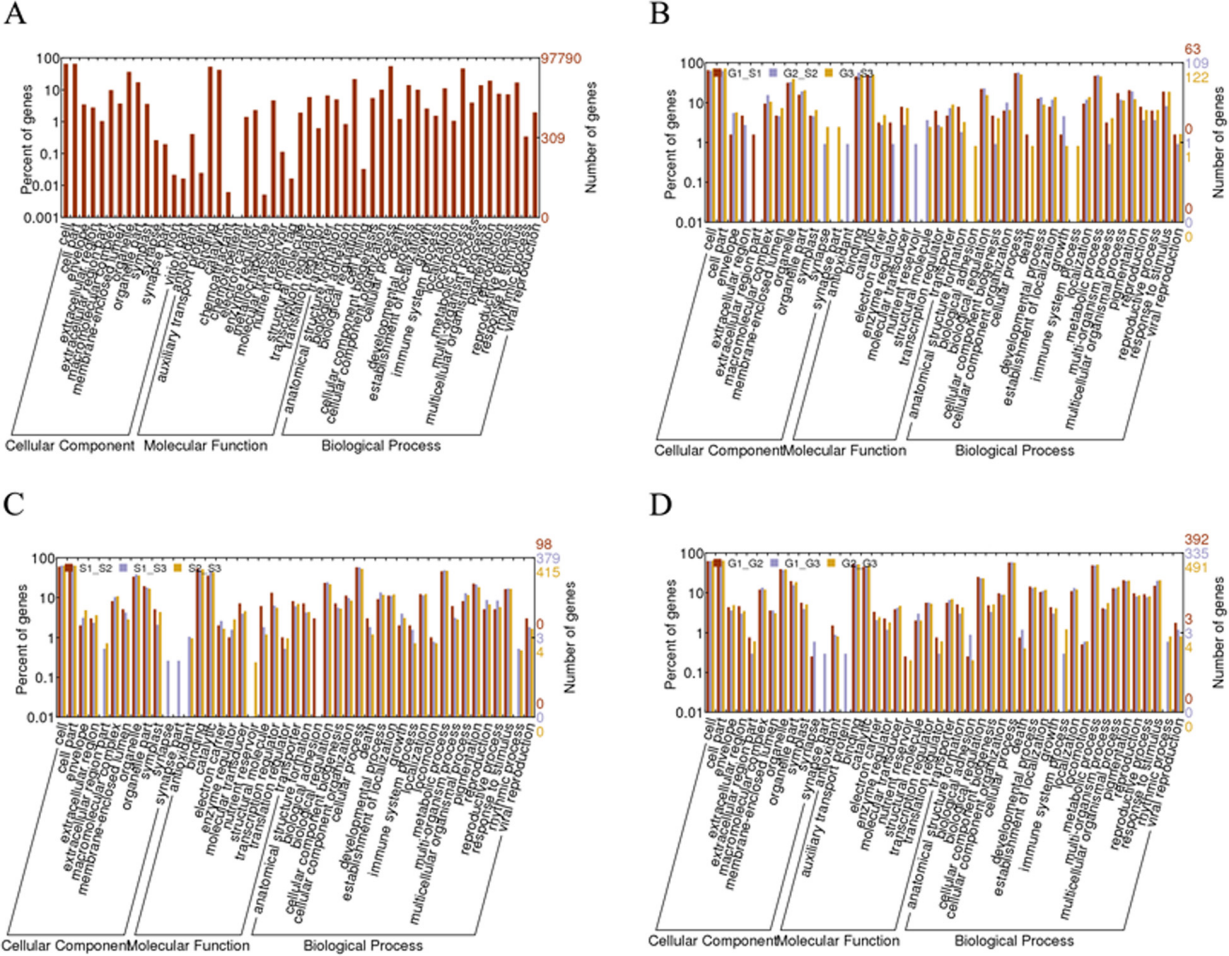
GO classifications of genes. Genes are divided into three main categories: biological process, molecular function and cellular component by GO analysis. **a** GO classification of all genes. **b** GO classification of DEGs among the two varieties at three stages. **c** GO classification among three contrasts (S1-S2, S1-S3, S2-S3) of Sollux. **d** GO classification among three contrasts (G1-G2, G1-G3, G2-G3) of Gaoyou

The Gene Ontology (GO) project provides an ontology of defined terms concerning gene product properties, which can promote our understanding on the gene function at the molecular level. The expressed genes could be classified into 53 GO subcategories. As shown in Fig. 3a, the GO category had three major terms: cellular component, molecular function and biological process. The proportions of genes assigned in the three terms were 26.4 %, 30.2 % and 43.4 %, respectively.

GO analysis of the DEGs between the three stages of the Sollux and of the Gaoyou showed 46 and 45 GO sub-categories for the two varieties (Fig. 3c and d), respectively. Different GO patterns were observed between the two varieties, for instance, the GO:0022610 (biological adhesion) only occurred in the comparison of S1-S2 for the Sollux, but in the comparison of G1-G2, G2-G3 and G1-G3 for the Gaoyou.

The candidate DEGs of S1-G1, S2-G2 and S3-G3 were grouped into 42 GO sub-categories (Fig. 3b) and 212 GO terms. GO analysis provided much insightful information regarding the GO terms. There was one GO sub-categories showed significant difference in the three comparisons, it’s GO:0050896 (response to stimulus). The *p* value based on gene percentage was below 0.05.

To know the significant terms of the DEG_GS_s (candidate DEGs between the Gaoyou and the Sollux), enrichment analysis was conducted against the background of GO term distribution of all expression genes. The significantly enriched GO terms of these DEG_GS_s were listed in Additional file 1: Table S1, Additional file 2: Table S2 and Additional file 3: Table S3. Terms with a variety of different functions enriched, in particular, GO terms such as lipid X metabolic process (GO:2001289) (Additional file 3: Table S3), lipid storage (GO:0019915) (Additional file 1: Table S1) and sphingolipid metabolic process (GO:0006665) (Additional file 3: Table S3) showed lipid-related function. These results represent an overall condition of the DEG_GS_s function; in particular, these lipid-related terms provide information to understand intervarietal oil content differences at gene function level.

### Metabolic pathway analysis

**Fig. 4:**
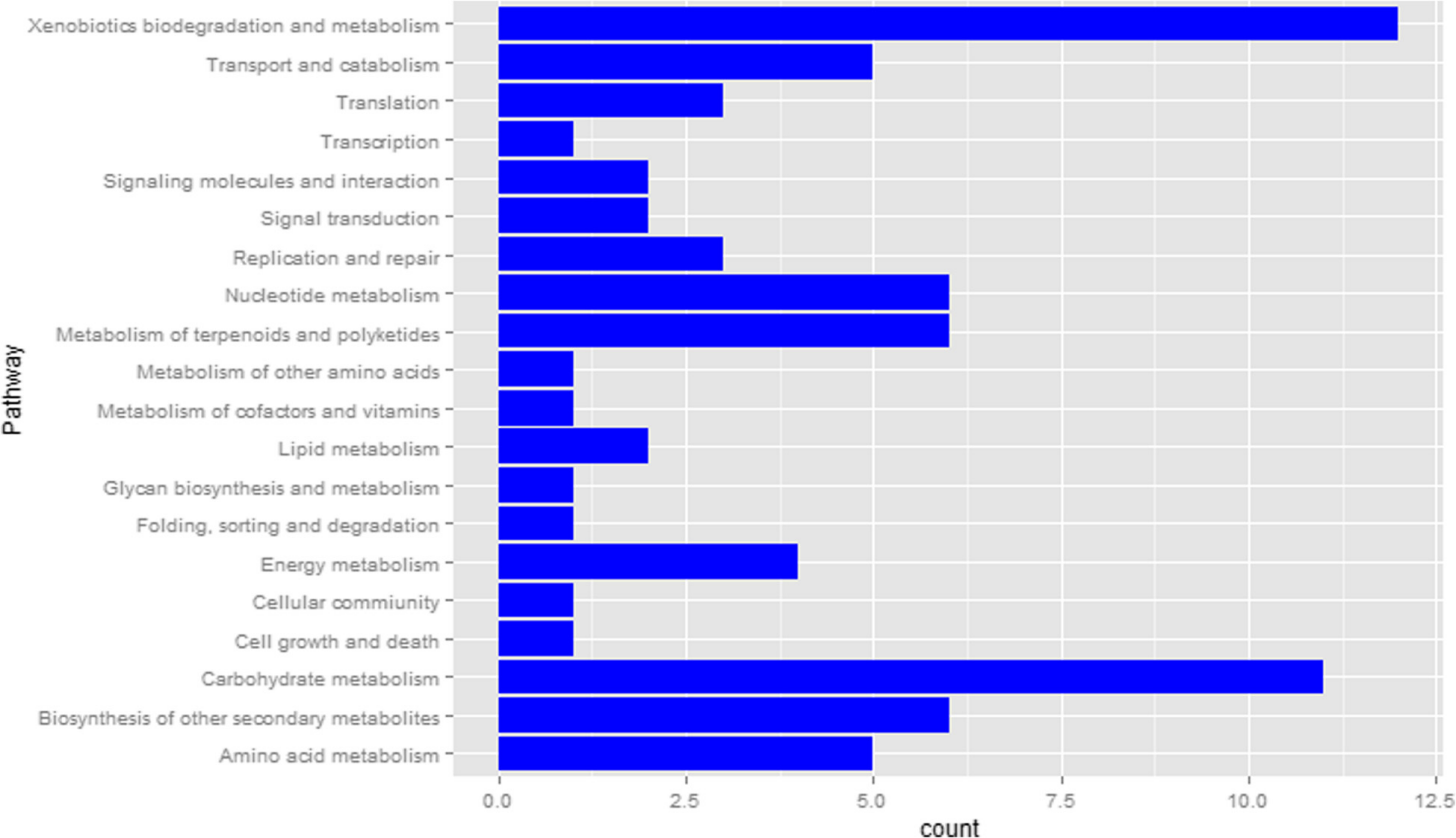
KEGG pathway categories of differentially expressed genes at 5–7 DAF, 15–17 DAF and 25–27 DAF

The information about metabolic pathways of the transcriptome is very valuable for understanding the physiological process of the pod after flowering. The predicted gene sequences were mapped to 12,480 KEGG orthology (KO) terms through the KAAS. To learn more about the differences between the two varieties, the metabolic pathways of DEGs were analyzed by classification. As a result, the DEGs at the three stages were grouped into 40 KO terms. These KO terms were classified into 20 functional categories (Fig. 4) and 59 branch pathways in total. The DEGs between the two varieties at 5–7 DAF, 15–17 DAF and 25–27 DAF were classified into 20, 21 and 25 branch pathways, respectively. As shown in Fig. 4, xenobiotics biodegradation and metabolism, and carbohydrate metabolism pathways account for large proportion of the DEG_GS_s. They were remarkable by enrichment test with *p* values of 8.4 × 10^−6^ and 0.028, respectively. Carbohydrate metabolism is important for the pod development, and then may affect the accumulation of other organics. Maybe lipid accumulation was also affected by it.

### Differentially expressed and lipid-related genes in the QTL regions

Nine significant QTLs of oil content were reported in the previous studies [15], named as OilA01, OilA05, OilA07, OilA09, OilC02, OilC03, OilC06, OilC08-1 and OilC08-2, which were located in linkage groups A01, A05, A07, A09, C02, C03, C06 and C08, respectively. The physical information of these QTLs is presented in Additional file 4: Table S4. DEG_GS_s and homologous genes to those involved in lipid metabolism were identified in the QTL regions by sequence alignment. Our analysis revealed a total of 33 DEG_GS_s in the QTL regions; the numbers of the DEG_GS_s located in the six QTLs (OilA01, OilA05, OilA09, OilC02, OilC03, OilC08-2) were 10, 3, 9, 3, 1 and 7, respectively (Table 3). 126 lipid-related homologous genes were discovered in the QTL regions. There were 4 and 35 lipid-related genes in OilC02 and OilC03 regions. 19 lipid-related genes resided in the OilC08-2, in which, there was one DEG_GS_. This lipid-related DEG is Sac-PIP (Sac domain-containing Phosphoinositide Phosphatase), which participates in phospholipid signaling pathway. In the OilC06 region, no DEG_GS_ was overlapped with the 11 lipid-related genes. 57 lipid-related homologous genes were located in the oil QTLs of A genome but did not overlap with DEG_GS_s. Further investigation of these genes is warranted.

**Table 3:** Number of genes, DEG_GS,_ and lipid-related homologous genes in the QTL region

QTL	Gene	DEG_GS_	Homologous
OilA01	1744	10	26
OilA05	1380	3	10
OilA07	322	0	4
OilA09	1331	9	17
OilC02	620	3	4
OilC03	1675	1	35
OilC06	894	0	11
OilC08-2	2798	7	19
OilC08-1	55	0	0

DEG_GS_ means differentially expressed genes between Gaoyou and Sollux. Homologous denotes genes homologous to lipid-related genes in *Arabidopsis thaliana*

Identification of the DEGs or the lipid-related homologous genes in the QTLs can substantially narrow down the number of candidate genes. The annotated functions of all these genes will provide better information. So, we listed all of the lipid-related genes (Additional file 5: Table S5) with their homologous gene names and the DEGs (Additional file 6: Table S6) with the gi numbers of their homologous genes in NR.

## Discussion

### Comparative analysis of gene expression between different stages and varieties

Pods of the *Brassica napus* play a key role in rapeseed growth. In addition to the protective function of encapsulating from pest and pathogens, the photosynthetically active pod wall contributes nutrients to fuel seed growth [16]. Thus, we used the pod to investigate the molecular mechanism of oil-related biological processes in three specific growth stages. To reduce the sequencing cost, we used the pooled samples to quantify the expression levels of the two varieties at the three growth stages. This cost-effective strategy would offer an affordable way to identify potential differentially expressed genes between the two varieties, although it could not provide a valid estimation of sampling and measurement errors as in individual sequencing. However, as in many other RNA-Seq studies that have no or very few replicates due to the still high cost of sequencing [17], our study did not have biological replicates. Therefore, it is still necessary to note that all the identified DEGs or DEG_GS_s are essentially candidates because of lack of biological replicates, although the FDR (false positive rate on DEG) could be alleviated or reduced by our pooled samples sequencing strategy to a certain extent.

The differential expression of a subset of genes can be identified through the comparative analysis of their transcriptomes. At the first stage (5–7 DAF), the least number of differentially expressed genes were detected and less than half of these DEGs had a higher expression in Gaoyou*.* Many of them are involved in the energy metabolism and carbohydrate metabolism. At the stage of 15–17 DAF, some of DEG_GS_s were found to be involved in the pathways of carbohydrate metabolism, amino acid metabolism, transportation and catabolism, and signal transduction, especially in the carbohydrate metabolism where 3 DEG_GS_s were involved. Among the DEG_GS_s, there is one related with lipid metabolism which was up-regulated in Gaoyou.

At 25–27 DAF, abundant DEG_GS_s were involved in some pathways, such as Xenobiotics biodegradation and metabolism, carbohydrate metabolism, biosynthesis of other secondary metabolites and metabolism of terpenoids and polyketides. Most of the Xenobiotics biodegradation and metabolism-related DEG_GS_s exhibited higher expression in the Sollux than in the Gaoyou.

In different growth periods of rapeseed, compared with the number of DEGs involved in different pathways, more DEGs participated in the pathway of ribosome (KO03010). Out of these DEGs involved in ribosome, 83 % in the Gaoyou and 58 % in the Sollux were up-regulated during three growth stages (from 5 to 7 DAF to 15–17 DAF, from 15 to 17 DAF to 25–27 DAF and from 5 to 7 DAF to 25–27 DAF). In cells, ribosome serves as the primary site of biological protein synthesis (i.e., translation). The expression pattern of the DEGs involved in ribosome indicates that protein synthesis increases with pod development.

Some DEGs involved in carbon fixation in photosynthetic organisms exhibited different expression patterns in the Sollux and in the Gaoyou. These genes were down-regulated from 5 to 7 DAF to 15–17 DAF and up-regulated from 15 to 17 DAF to 25–27 DAF in Sollux. In Gaoyou, these genes were down-regulated from 5 to 7 DAF to 15–17 DAF, and also down-regulated in expression from 15 to 17 DAF to 25–27 DAF. It indicates that the Gaoyou and the Sollux have different active periods of carbon fixation in pod.

### Lipid-related genes in pod development after flowering

Rapeseed contains various components and many genes are involved in different metabolic pathways to participate in the formation of oil content. Genes homologous to acyl lipids-related genes from ARABIDOPSIS ACYL-LIPID METABOLISM database were analyzed. In the Sollux, there was only one lipid-related DEG between S1 and S2, while 10 and 9 were detected between S1 and S3, and between S2 and S3, respectively, most of which were up-regulated at the later stage. Six lipid-related DEGs with high expression values were *PI5P-II, CDP-DAGS, LPLA, KdtA, PI3P* and *PIPK-IA.* Apart from *PI5P-II,* the others were up-regulated. The *PI5P-II, LPLA, PI3P* and *PIPK-IA* are involved in the phospholipid signaling pathway, and the *CDP-DAGS* and *KdtA* are involved in the Mitochondrial Phospholipid Synthesis and Mitochondrial Lipopolysaccharide Synthesis. During the growth stage of the Gaoyou, 8 out of 10 differentially expressed lipid-related genes were up-regulated during the G1-G2. The numbers of lipid-related DEGs were 10 and 9 during the G2-G3 and G1-G3, respectively, and 4 and 1 DEGs were down-regulated, respectively (Table 4 and Additional file 7: Table S7).

**Table 4:** The differentially expressed oil-related homologous genes in the two varieties

	Up-regulated	Down-regulated
S1-S2	FAH	
S2-S3	PIPK-IA, CDP-DAGS, PI3P, FAH, PDAT, MAGL, LPLA	PI5P-II, FAD8
S1-S3	PIPK-IA, CER3, CDP-DAGS, CYP450, PDAT, At3g61150	PI5P-II
	KdtA, PI4K, LPLA	
G1-G2	CER3, PDAT, HAD, At5g66450, FAD8,KdtA, STERO	PI4K, DOP
	PGPS	
G2-G3	PIPK-IA, PI3P, FAH, OBO, PDAT, MAGL	PI5P-II,PLAT, HAD, FAD8,
G1-G3	HmACC, CER3, BC, At5g66450, Sac-PIP, KdtA, OBO, STERO	DOP

S and G denote the Sollux and the Gaoyou, respectively, and the numbers 1–3 denote the three stages, respectively; The information of DEGs and corresponding oil-related homologous genes in Arabidopsis are listed in Additional file 7: Table S7

The Gaoyou performs better than the Sollux for the oil content in Hangzhou, China, which can be partly explained by the transcriptional level in our study. The result indicated that vast majority of the lipid-related genes exhibited no difference in expression level between the two varieties. At the stages of 5–7 DAF, there was no significant lipid-related DEG between the Gaoyou and the Sollux. Eight lipid-related DEGs were detected at 15–17 DAF and 25–27 AF. Out of the eight DEGs, three genes (*MCMT, OBO, Sac-PIP*) exhibited a higher expression level in the Gaoyou. The cellular function of *OBO* is related to oil bodies, which plays an important role in the TAG synthesis [18]. Therefore, the expression level of *OBO* could affect the final size of oil body. *MCMT* (Malonyl-CoA) plays a key role in chain elongation in fatty acid biosynthesis and polyketide biosynthesis [19], as it provides 2-carbon units to fatty acids and commits them to fatty acid chain synthesis. *Sac-PIP* (Sac domain-containing Phosphoinositide Phosphatase) participates in phospholipid signaling pathway. Other five DEGs (*GPI-PLC, PI5P-II, At3g13900, MFP, PDAT*) had higher expression level in *Sollux*. Both *PI5P-II* and *GPI-PLC* are related to phospholipid signaling which is crucial for plant growth and development [20]. At3g13900 is a putative phospholipid-transporting ATPase that cannot be classified into a certain pathway. The aforementioned three genes that had a higher expression in Sollux had no a direct correlation between gene function and the lipid synthesis/degradation. *MFP*, a multifunctional protein, can catalyze four separate reactions, two of which play an important role in the catabolism of all fatty acids [21].

On the basis of the above results, we conclude that the expression pattern of lipid-related genes may result in better performance in character of oil content for the Gaoyou under the cultivation condition in Hangzhou, China.

### Combining significant DEGs and QTLs for oil content

The *Arabidopsis thaliana* lipid-related homologous genes were mapped to the *Brassica napus*, of which some lipid-related candidate loci reside in the regions of these QTLs. Benefiting from identification of more genes involved in acyl-lipid metabolism in *Arabidopsis*, more lipid-related candidate genes could be identified in the QTL regions by sequence alignment. According to the genome annotation of the Chinese cabbage (*Brassica rapa*), a total of 10,819 genes were found in the regions of the nine QTLs, whereas it is still a challenge to screen lipid-related candidate genes. Through the comparative analysis with *Arabidopsis* lipid-related genes, candidate lipid-related genes in the region of QTLs of the *Brassica napus* can be efficiently distinguished. Integration of the homologous, QTL and expression information is the directly and efficiently way to identify the candidate genes which are quite significant for our study.

Most of the *Arabidopsis* lipid-related homologous genes are not associated with the QTLs; it is probably due to the complexity in the genetic architecture of seed oil content and/or the limited knowledge on gene annotation. Another potential reason is that only limited QTLs harboring major-effect genes are detected in QTL mapping because of insufficient power. Most DEG_GS_s in the QTL regions are not homologous to the lipid-related genes of *Arabidopsis*. The annotation of these genes was achieved by sequence alignment between the predicted protein sequence and the NR database. A subset of the genes are functionally related to the oil content, whereas the others have no clear relationship with the lipid-related pathways. In OilA1, there is one DEG_GS_ homologous to the GDSL esterase/lipase with function in hydrolase activity. It is located in the endomembrane system and involved in lipid metabolic process [22]. Further study on the other DEG_GS_s in these QTL regions that have no straightforward relationship with the seed oil content are needed for understanding their potential roles and importance in the oil content related metabolic pathways. These continuing works will provide valuable resources for breeding program of the *Brassica napus*.

## Methods

### Plant materials and RNA extraction

The two parents of the Sollux/Gaoyou doubled haploid (DH) population (Zhao et al., 2005), Sollux and Gaoyou, were used for transcriptome profiling in this study. The Sollux is a typical German winter rapeseed cultivar and the Gaoyou is an inbred line from Chinese variety Gaoyou 605 with no vernalization requirement. While both parents have high oil content in seeds and do not show large difference in oil content, there are large inherited differences in many morphological traits. In the six years mapping experiments in Hangzhou, China [15], the Gaoyou had higher oil content and better agronomic performance than the Sollux. There was a transgressive segregation phenomenon where lower or higher oil content beyond the parents could be observed in the DH population, potentially ascribable to recombination of functional genes underlying oil content (Table 5), suggesting a genetic difference in oil content between the parents. The differences between the lines with the highest and lowest oil contents were averagely up to 11.5 % over the six years mapping experiments.

**Table 5:** Phenotypic variation of seed oil content (%) in the Sollux/Gaoyou DH population and the parents

Year	2001	2004	2005	2007	2008	2009
Min	38.3	35.6	38.5	41.7	43.8	45.0
Max	49.5	48.9	52.7	51.4	53.3	56.4
Sollux	41.6	41.7	43	41.2	48.2	47.9
Gaoyou	44.7	43.8	46.7	49.5	49.3	52.5

2001, 2004, 2005, 2007, 2008 and 2009 indicate the years to conduct experiments in Hangzhou, China; Min and Max indicate the minimum and the maximum of observation values in the DH population derived from the crossing between the Sollux and the Gaoyou

Sollux and Gaoyou were planted in the experimental farm of Zhejiang Academy of Agricultural Sciences for this study in 2011. Gaoyou plants were first grown in growth chamber (<10 °C, 16 h light) during seedling stage and moved into greenhouse when getting the same bolting stage as Sollux. Total RNA of two cultivars were extracted from pods using Trizol reagent (Invitrogen, Carlsbad, CA, USA) at the three different stages: 5–7 DAF, 15–17 DAF and 25–27 DAF, respectively, and were stored in liquid nitrogen at the temperature of −80 °C. The RNA was purified using RNeasy Plant Mini Kit (Qiagen, Valencia, CA). The quality was verified using a 2100 Bioanalyzer RNA Nanochip (Agilent, Santa Clara, CA), and the RNA Integrity Number (RIN) of all the samples were more than 8.5. The RNA samples from six plants of each variety at each stage were pooled for RNA-Seq.

### cDNA library construction and sequencing

Sequence libraries were prepared according to the manufacturer’s instructions (Illumina, San Diego, CA). Poly-A-containing mRNA was isolated from the total RNA, subjected to two purification rounds using poly-T oligo-attached magnetic beads, and fragmented using divalent cations at 94 °C. Taking these fragments as templates, the first-strand cDNA was synthesized using random hexamer primers and Super-script TM III (Invitrogen TM, Carlsbad, CA, USA). Following the second strand cDNA synthesis and adaptor ligation, 200-bp and 50-bp cDNA fragments were isolated using gel electrophoresis and amplified by 18 cycles of PCR. After the amplification, the libraries were sequenced using Illumina Hiseq 2000. The adaptor sequences, empty reads and low quality sequences were filtered from the raw reads.

### Gene annotation and bioinformatics analysis

Bowtie indexes the reference sequences with a Burrows-Wheeler index which can keep its memory footprint small [23]. Tophat is a fast mapping tool for RNA-seq reads that can identify splice junction between exons [24]. Cufflinks can assembly the reads into transcripts based on the mapping results [25]. Tophat v2.0.12 and Bowtie v2.2.3 were used to align the high quality reads of six samples (S1, S2, S3, G1, G2, and G3) to the *Brassica napus* genome sequences [26]. Then Cufflinks was used to assembly the transcripts of each sample. In addition to Cufflinks, there are several other softwares such as IsoEM that can be used to infer isoform and gene expression levels from high-throughput transcriptome sequencing [27, 28], and MaLTA [28] that can be used to transcriptome assembly and quantification from Ion Torrent RNA-Seq data.

The *Brassica napus* genome annotation downloaded from the NCBI database was merged with the annotation obtained from Cufflinks by cuffmerge. The predicted genes from the merged genome annotation were alignment with the plant part of NR database by blastx 2.2.26+ with evalue less than 1E-5. So the gene functions can be obtained from homologous genes in the NR database.

GO annotation were performed by alignment between genes and GO database. Website tool WEGO (http://wego.genomics.org.cn/cgi-bin/wego/index.pl) [29] was used to produce GO functional classification of all the expressed genes and to interpret the distribution of gene functions at the macro level. The GO enrichment analysis of the DEG_GS_s (DEG between the Gaoyou and the Sollux) was performed by hypergeometric test in R. R function dhyper was used to calculate P-values, the GO terms with P-value less than 0.01 were considered to be enrichment terms.

At last, the predicted genes were submitted to the KAAS (http://www.genome.jp/tools/kaas/) [30] website to obtain KEGG annotations. As same as the GO enrichment analysis, the kegg enrichment analysis was also performed.

Cuffdiff was used to identify DEGs [25, 31–33]. The DEGs were filtered using a p value cut off of 0.01 and a false discovery rate below 0.05 [14]. It is also helpful for us to measure gene expression level by calculating FPKM, similar to RPKM (reads per kilobase of gene model exon, per million mapped reads) which is used earlier [34]. As genes with similar expression patterns are often functionally correlated, to identify such clusters, the hierarchical clustering of DEGs between the two varieties at the three stages was performed using R package gplots (http://cran.r-project.org/web/packages/gplots/index.html).

### Analysis of genes involved in QTL regions

Nine significant QTLs for oil content in *Brassica napus* have been detected in the previous studies, four of which are located in A genome, and the other five are located in C genome. The parents of the mapping population in that study are the same as used in the present study. The genome sequence of *Brassica napus* were downloaded from the *Brassica napus* database (www.genoscope.cns.fr/brassicanapus). In order to distinguish genes homologous to the genes involved in lipid metabolism collected in ARABIDOPSIS ACYL-LIPID METABOLISM (http://aralip.plantbiology.msu.edu/pathways/pathways), sequence alignment was performed between genes in the QTL regions and the lipid-related genes were identified by blastn with evalue less than 10^−5^.

## Conclusions

This study provided a comprehensive overview on the pod transcriptomes of two varieties with different oil-contents at the three developmental stages. Integrated analysis of transcriptome profiling, QTL mapping and comparative genomics with other relative species leads to efficient identification of most plausible functional genes underlying oil-content related characters, offering valuable resources for bettering breeding program of *Brassica napus*.

## Additional files

Additional file 1: Table S1: The top 20 most represented GO terms of DEG in the biological process category at the 5–7 DAF (S1-G1). (XLS 29 kb) Additional file 2: Table S2: The top 20 most represented GO terms of DEG in the biological process category at the 15–17 DAF (S2-G2). (XLS 33 kb) Additional file 3: Table S3: The top 20 most represented GO terms of DEG in the biological process category at the 25–27 DAF (S3-G3). (XLS 32 kb) Additional file 4: Table S4: The physical location of the oil related QTLs. (XLS 21 kb) Additional file 5: Table S5: The lipid-related homologous genes in arabidopsis for the genes in QTL regions. (XLS 24 kb) Additional file 6: Table S6: The gi number of genes in NR database homologous with DEGs between varieties in QTL regions. (XLS 25 kb) Additional file 7: Table S7: Lipid-related DEGs in *Brassica napus* and their lipid-related homologous genes in arabidopsis. (XLS 33 kb)

## References

[1] Ecke W, Uzunova M, Weissleder K (1995). Mapping the genome of rapeseed (Brassica napus L.). II. Localization of genes controlling erucic acid synthesis and seed oil content. Theor Appl Genet.

[2] Qiu D, Morgan C, Shi J, Long Y, Liu J, Li R (2006). A comparative linkage map of oilseed rape and its use for QTL analysis of seed oil and erucic acid content. Theor Appl Genet.

[3] Zhao J, Becker HC, Zhang D, Zhang Y, Ecke W (2006). Conditional QTL mapping of oil content in rapeseed with respect to protein content and traits related to plant development and grain yield. Theor Appl Genet.

[4] Farh KK, Marson A, Zhu J, Kleinewietfeld M, Housley WJ, Beik S (2015). Genetic and epigenetic fine mapping of causal autoimmune disease variants. Nature.

[5] Li RJ, Wang HZ, Mao H, Lu YT, Hua W (2006). Identification of differentially expressed genes in seeds of two near-isogenic Brassica napus lines with different oil content. Planta.

[6] Vigeolas H, Waldeck P, Zank T, Geigenberger P (2007). Increasing seed oil content in oil-seed rape (Brassica napus L.) by over-expression of a yeast glycerol-3-phosphate dehydrogenase under the control of a seed-specific promoter. Plant Biotechnol J.

[7] Katavic V, Friesen W, Barton DL, Gossen KK, Giblin EM, Luciw T (2000). Utility of the Arabidopsis FAE1 and yeast SLC1-1 genes for improvements in erucic acid and oil content in rapeseed. Biochem Soc Trans.

[8] Badapanda C (2013). Suppression subtractive hybridization (SSH) combined with bioinformatics method: an integrated functional annotation approach for analysis of differentially expressed immune-genes in insects. Bioinformation.

[9] Wang Z, Gerstein M, Snyder M (2009). RNA-Seq: a revolutionary tool for transcriptomics. Nat Rev Genet.

[10] Trick M, Long Y, Meng J, Bancroft I (2009). Single nucleotide polymorphism (SNP) discovery in the polyploid Brassica napus using Solexa transcriptome sequencing. Plant Biotechnol J.

[11] Higgins J, Magusin A, Trick M, Fraser F, Bancroft I (2012). Use of mRNA-seq to discriminate contributions to the transcriptome from the constituent genomes of the polyploid crop species Brassica napus. BMC Genomics.

[12] Pandit A, Rai V, Bal S, Sinha S, Kumar V, Chauhan M (2010). Combining QTL mapping and transcriptome profiling of bulked RILs for identification of functional polymorphism for salt tolerance genes in rice (Oryza sativa L.). Mol Genet Genomics.

[13] Zhao JY, Becker HC, Ding HD, Zhang YF, Zhang DQ, Ecke W (2005). QTL of three agronomically important traits and their interactions with environment in a European x Chinese rapeseed population. Yi Chuan Xue Bao.

[14] Ness RW, Siol M, Barrett SC (2011). De novo sequence assembly and characterization of the floral transcriptome in cross- and self-fertilizing plants. BMC Genomics.

[15] Zhao J, Huang J, Chen F, Xu F, Ni X, Xu H (2012). Molecular mapping of Arabidopsis thaliana lipid-related orthologous genes in Brassica napus. Theor Appl Genet.

[16] Bennett EJ, Roberts JA, Wagstaff C (2011). The role of the pod in seed development: strategies for manipulating yield. New Phytol.

[17] Al SS, Tiagueu YT, Zelikovsky A, Mandoiu II (2014). Bootstrap-based differential gene expression analysis for RNA-Seq data with and without replicates. BMC Genomics.

[18] Kim HU, Hsieh K, Ratnayake C, Huang AH (2002). A novel group of oleosins is present inside the pollen of Arabidopsis. J Biol Chem.

[19] Bryant N, Lloyd J, Sweeney C, Myouga F, Meinke D (2011). Identification of nuclear genes encoding chloroplast-localized proteins required for embryo development in Arabidopsis. Plant Physiol.

[20] Xue HW, Chen X, Mei Y (2009). Function and regulation of phospholipid signalling in plants. Biochem J.

[21] Rylott EL, Eastmond PJ, Gilday AD, Slocombe SP, Larson TR, Baker A (2006). The Arabidopsis thaliana multifunctional protein gene (MFP2) of peroxisomal beta-oxidation is essential for seedling establishment. Plant J.

[22] Mayer K, Schuller C, Wambutt R, Murphy G, Volckaert G, Pohl T (1999). Sequence and analysis of chromosome 4 of the plant Arabidopsis thaliana. Nature.

[23] Langmead B, Trapnell C, Pop M, Salzberg SL (2009). Ultrafast and memory-efficient alignment of short DNA sequences to the human genome. Genome Biol.

[24] Trapnell C, Pachter L, Salzberg SL (2009). TopHat: discovering splice junctions with RNA-Seq. Bioinformatics.

[25] Roberts A, Pimentel H, Trapnell C, Pachter L (2011). Identification of novel transcripts in annotated genomes using RNA-Seq. Bioinformatics.

[26] Chalhoub B, Denoeud F, Liu S, Parkin IA, Tang H, Wang X (2014). Plant genetics. Early allopolyploid evolution in the post-Neolithic Brassica napus oilseed genome. Science.

[27] Nicolae M, Mangul S, Mandoiu II, Zelikovsky A (2011). Estimation of alternative splicing isoform frequencies from RNA-Seq data. Algorithms Mol Biol.

[28] Mangul S, Caciula A, Al SS, Brinza D, Mndoiu I, Zelikovsky A (2014). Transcriptome assembly and quantification from Ion Torrent RNA-Seq data. BMC Genomics.

[29] Ye J, Fang L, Zheng H, Zhang Y, Chen J, Zhang Z (2006). WEGO: a web tool for plotting GO annotations. Nucleic Acids Res.

[30] Moriya Y, Itoh M, Okuda S, Yoshizawa AC, Kanehisa M (2007). KAAS: an automatic genome annotation and pathway reconstruction server. Nucleic Acids Res.

[31] Trapnell C, Hendrickson DG, Sauvageau M, Goff L, Rinn JL, Pachter L (2013). Differential analysis of gene regulation at transcript resolution with RNA-seq. Nat Biotechnol.

[32] Roberts A, Trapnell C, Donaghey J, Rinn JL, Pachter L (2011). Improving RNA-Seq expression estimates by correcting for fragment bias. Genome Biol.

[33] Trapnell C, Williams BA, Pertea G, Mortazavi A, Kwan G, van Baren MJ (2010). Transcript assembly and quantification by RNA-Seq reveals unannotated transcripts and isoform switching during cell differentiation. Nat Biotechnol.

[34] Mortazavi A, Williams BA, McCue K, Schaeffer L, Wold B (2008). Mapping and quantifying mammalian transcriptomes by RNA-Seq. Nat Methods.

